# Valorization Potentials of Rapeseed Meal in a Biorefinery Perspective: Focus on Nutritional and Bioactive Components

**DOI:** 10.3390/molecules26226787

**Published:** 2021-11-10

**Authors:** Gabriella Di Lena, Jose Sanchez del Pulgar, Massimo Lucarini, Alessandra Durazzo, Petra Ondrejíčková, Florin Oancea, Rodica-Mihaela Frincu, Altero Aguzzi, Stefano Ferrari Nicoli, Irene Casini, Paolo Gabrielli, Roberto Caproni, Igor Červeň, Ginevra Lombardi-Boccia

**Affiliations:** 1 CREA Research Centre for Food and Nutrition, Via Ardeatina 546, 00178 Rome, Italy; jsapuri@hotmail.com (J.S.d.P.); massimo.lucarini@crea.gov.it (M.L.); alessandra.durazzo@crea.gov.it (A.D.); altero.aguzzi@crea.gov.it (A.A.); stefano.nicoli@crea.gov.it (S.F.N.); irene.casini@crea.gov.it (I.C.); paolo.gabrielli@crea.gov.it (P.G.); roberto.caproni@crea.gov.it (R.C.); g.lombardiboccia@crea.gov.it (G.L.-B.); 2 ENVIRAL a.s., Trnavská Cesta, 920 41 Leopoldov, Slovakia; Ondrejickova@enviengroup.eu; 3 National Institute for Research and Development in Chemistry and Petrochemistry—ICECHIM, 060021 Bucharest, Romania; florin.oancea@icechim.ro (F.O.); icechim.calarasi@gmail.com (R.-M.F.); 4 Poľnoservis a.s., Trnavská Cesta, 920 41 Leopoldov, Slovakia; cerven@polnoservis.sk

**Keywords:** rapeseed meal, agri-food by-products, valorization, biorefinery, chemical characterization, nutrients, phenolic compounds, bioactive compounds, antioxidant activity

## Abstract

Rapeseed meal (RSM), a by-product of oilseed extraction connected to the agri-food and biofuel sectors, is currently used as animal feed and for other low-value purposes. With a biorefinery approach, RSM could be valorized as a source of bio-based molecules for high-value applications. This study provides a chemical characterization of RSM in the perspective of its valorization. A qualitative study of main functional groups by fourier transform infrared (FTIR) spectroscopy was integrated with a chemical characterization of macronutrients, minerals by inductively coupled plasma optical emission spectrometry (ICP-OES), phenolic acids and lipid components by high performance liquid chromatography-tandem mass spectrometry (HPLC-MS/MS), HPLC-diode-array detector (HPLC-DAD) and gas chromatography-mass spectrometry/flame ionization detector (GC-MS/FID). The study, conducted on different lots of RSM collected over a one-year period from an oil pressing factory serving a biofuel biorefinery, highlighted a constant quality over time of RSM, characterized by high protein (31–34%), fiber (33–40%) and mineral (5.5–6.8%) contents. Polyphenol extracts showed a significant antioxidant activity and a prevalence of sinapic acid, accounting for more than 85% of total phenolic acids (395–437 mg kg^−1^ RSM). Results highlight the potentialities of RSM for further valorization strategies that may lead to the creation of new cross-sector interconnections and bio-based value chains with improvement of the economics and sustainability of the bioeconomy sectors involved.

## 1. Introduction

Rapeseed is the second most cultivated oilseed crop worldwide after soybean, with a production accounting for 68 million tons in 2020, providing a global volume of nearly 28 million metric tons of rapeseed oil worldwide [1]. In Europe, rapeseed is the dominant oilseed crop and its production accounts for almost 25% of the global production, with France, Germany and Poland as the top producing countries.

The rapeseed market in Europe is mainly driven by the demand for rapeseed oil, which is mostly used by the food and feed industries and, to a lesser extent, by the biodiesel industry sector [2].

In Europe, rapeseed is the main feedstock for biodiesel production, a sector highly dependent on biofuel policies and regulations aiming at the diversification of energy supplies, the increment of competitivity and sustainability of biofuels and the reduction of greenhouse gas emissions [3,4,5]. Furthermore, the European strategy for the promotion of protein crops encourages the production of protein-rich plants, including rapeseed, to reduce dependence on imported vegetable proteins (mainly soy from third countries) and accelerate the transition to more sustainable agri-food and farming systems [6].

The growth of rapeseed oil production coincides with a proportional production of a significant co-stream in the form of press cake or meal. This co-product, resulting after subsequent oil extractions from rapeseeds after the pressing process, is produced in large quantities worldwide (40 million tons/year) and in the EU (12.5 million tons in 2020), [7]. 

With the depletion of natural resources, increment of greenhouse gas emissions and growing awareness of the need for sustainable development and valorization of agro-industrial by-products and wastes, the transformation of biomass into valuable materials and energy is a global emerging concern [8,9].

Currently used as a protein complement in animal feed mixtures and sometimes as fertilizer or as combustible source, rapeseed meal should be valorized for higher end-uses, being an excellent source of nutritionally valuable and bioactive molecules. Rapeseed meal contains proteins with a well-balanced amino acid composition [10]; it may therefore represent a good alternative protein source to meet the growing global demand for protein expected in the next decades. 

However, despite its great nutritional potential, the use of rapeseed meal in the food and feed sectors is limited by the presence of antinutritional factors. While the development of canola varieties, with low glucosinolate and erucic acid contents, have partially solved these problems, the high content of phenolic compounds and the presence of phytates and fiber affect nutrient bioavailability and sensory and functional properties. In particular, one issue related to rapeseed is the antinutritional effect of polyphenols [11,12]. These molecules deserve not to be discarded, but valorized because of their strong antioxidant, antimicrobial and health-promoting properties [13]. One strategy to recover several components co-existing in rapeseed meal and to preserve their native properties may be the application of separation technologies. 

With a biorefinery approach, rapeseed meal could be exploited as an excellent source of valuable nutrients and bioactive compounds to develop innovative bio-based products for the pharmaceutical, nutraceutical, cosmetic, food and feed sectors.

Since the production of rapeseed in the EU reached 16.2 million tons in 2020 [7] and is expected to grow in the coming decades, the full valorization of rapeseed meal through integrated biorefining represents a real breakthrough for the biofuel and agri-food markets. One issue related to the valorization of rapeseed meal through the biorefinery is the variability of its composition due to various cultivars, soil, and climatic conditions. The knowledge regarding this variability is still scarce, especially for the rapeseed sourced from central and eastern Europe, and needs further investigation.

In this paper we report the results of a study focused on the assessment of components of nutritional, cosmeceutical and nutraceutical interest (macronutrients, minerals, phenolic acids and lipid components) in rapeseed meal obtained over a 1-year period by an oil processing plant serving a biofuel biorefinery. Moreover, the qualitative study of main functional groups of components in rapeseed meal by fourier transform infrared (FTIR) spectroscopy and the assessment of the antioxidant activity of extracts were carried out. The aim of this study is to give a comprehensive picture of the potentials, in a biorefinery perspective, of a side-stream of interest to two key-players in the bioeconomy, the biofuel and the agri-food sectors, as a source of bio-based molecules for high-value market products.

## 2. Results and Discussion

### 2.1. Qualitative Analysis of Main Functional Groups: The Fourier Transform Infrared-Attenuated Total Reflection (FTIR-ATR) Approach

Different studies have successfully used FTIR for characterizing waste and biomass [14,15,16,17,18]. On the other hand, FTIR-ATR could be considered as an innovative, green and rapid methodology, having the advantages to require a minimum or no sample preparation and also allowing a rapid characterization of samples. 

FTIR spectroscopy can be defined as a “fingerprint analytical technique” for the structural identification of compounds considering that no two chemical structures will have the same FTIR spectrum [19]. FTIR provides a characteristic signature of chemical or biochemical substances present in the sample by featuring their molecular vibrations (stretching, bending, and torsions of the chemical bonds) in specific infrared regions.

**Figure 1:**
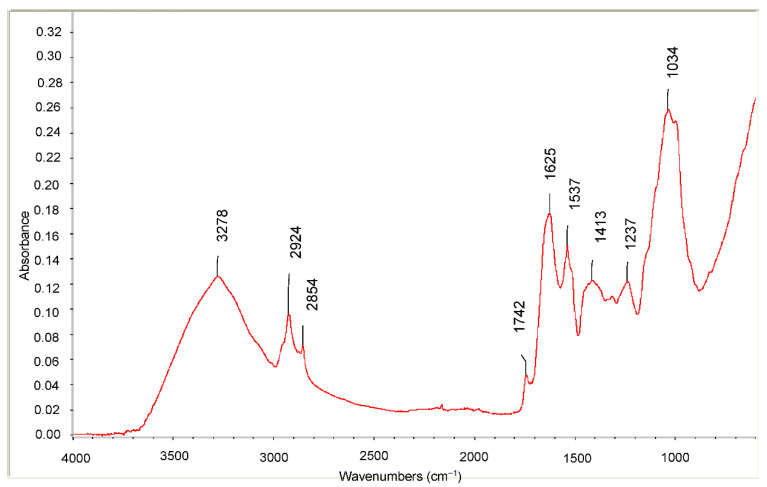
The averaged spectra from the rapeseed meal samples.

The averaged spectra from the rapeseed meal samples are shown in Figure 1.

In the spectra displayed, the specific bands characteristic of the rapeseed meal samples are highlighted and assigned as follows. The broad band peaking at around 3278 cm^−1^ corresponds to the OH stretching modes. It can be attributed to the polysaccharides and/or cellulose [20,21]. Asymmetric and symmetric stretching vibrations of CH_2_ groups are found at 2924 and 2854 cm^−1^, respectively. They are mainly associated with the hydrocarbon chains like those of fatty acids. The spectral band at 1742 cm^−1^ is attributed to the absorption of the C=O bonds of the ester groups and it is related to the presence of the fatty acids and their glycerides, as well as pectins and lignin [22]. The amide I band at 1625 cm^−1^ results from the C=O stretching the amides I, II, and III (carbonyl stretching vibrations of the peptide backbone) [23], while the amidic band II at 1537 cm^−1^ originates from the bending vibrations of the N-H groups. The fingerprint region from 1500 to 800 cm^−1^ represents a region rich in peaks derived from several bending, stretching, scissoring, rocking and torsional modes. Even if this region is rich in information, it is very difficult to analyze because of its complexity. Key information on organic compounds, i.e., sugars, alcohols, and organic acids, are given by the analysis of this area. The aromatic C-C stretching 1413 cm^−1^ is related to the presence of phenolic compounds. The C-O stretching at ~1034 cm^−1^ are due to polysaccharide structures.

### 2.2. Macronutrients and Minerals

The chemical composition of rapeseed meal collected from the oil pressing and processing plant in the period July 2018–September 2019 is reported in Table 1. 

Quality checks routinely accomplished on a daily basis at the oil-pressing factory was confirmed by analyses at CREA. Rapeseed meal was characterized by high fiber (33–40%) and protein (31–34%) contents. Carbohydrates (6–13%) and ashes (5.5–6.8%) were present at lower levels along with very low residual amounts of lipids (1.4–3.3%). Quantification of soluble and insoluble dietary fiber fractions indicated a prevalence of insoluble fiber (87%), mainly consisting of cellulose and lignin, over soluble fiber (13%), composed of hemicellulose and pectins. The protein value, a parameter routinely monitored at the factory because of its importance in the subsequent feed application, was quite constant and in compliance with the values required for livestock feed mixtures.

The composition of the different lots of rapeseed meal examined over the specified period of time showed a low variability, indicating a comparable quality of the feedstock over time and standardized industrial processing conditions during the pressing process and subsequent oil extraction and drying. The slight compositional variations detected, connected to different pedo-climatic conditions, specific harvest time, storage conditions and duration within the year, are an important requisite in a biorefinery perspective, since a constant chemical composition of the feedstock helps a standardization of downstream processes.

The high protein content in rapeseed, together with its balanced amino acid composition and high quantities of essential amino acids, particularly sulphur-containing ones, make rapeseed a promising source of sustainable proteins, providing ingredients for novel food products [24,25]. The two major storage proteins, cruciferins and napins, are reported to account altogether for 80–90% of the total protein content of rapeseed and have different physico-chemical and functional properties [26].

Canola proteins are also considered an attractive source of bioactive peptides as they may be converted through enzymatic hydrolysis into several different peptide fractions with anti-hypertensive and antioxidant properties, bile-acid binding capacity and anti-thrombotic activity [27,28,29,30]. In addition, native canola proteins and their hydrolysates are characterized by interesting functional properties, especially water-binding, foaming and gel-forming capacities, that render them a candidate ingredient in different food formulations in replacement of animal proteins [25].

Based on its high fiber content and with respect to the physiological importance of fiber in the human diet, rapeseed meal could be considered a feasible, low-cost source of this functional ingredient for the food industry. Nevertheless, for rapeseed meal as well as other oilseed cakes, the recovery of fiber fractions as value-added ingredients for the food industry is still an undeveloped field [31]. However, as the market of bakery and breakfast products is under constant search for innovative ingredients, a possible direct application of rapeseed meal as a functional food ingredient by virtue of its composition is being considered [32,33].

The mineral and trace element profile of rapeseed meal showed high amounts of potassium, phosphorus, calcium, magnesium and low amounts of sodium (Table 2), in line with common vegetable feedstocks. Among trace elements, of particular interest is the iron content, which ranged from 69.23 to 106.61 mg kg^−1^ dry basis, as well as the zinc content, which was in the range of 49.20–57.13 mg kg^−1^ dry basis. These high values compared to cereal flours (i.e., white, plain, soft wheat flour: Fe 1.94 mg 100 g^−1^ edible portion, Zn 0.7 mg 100 g^−1^ edible portion; wholemeal wheat flour: Fe 2.47 mg 100 g^−1^ edible portion, Zn 1.9 mg 100 g^−1^ edible portion [34]) suggest rapeseed meal as a potential functional ingredient in blend flours. 

One limiting factor to the performance of rapeseed/canola proteins according to literature is the presence of phytates (in particular myo-inositol hexaphosphate, IP_6_, and pentaphosphates, IP_5_) that can strongly bind minerals like calcium, zinc, iron and copper, reducing their bioavailability. On the other hand, processing can reduce IP_6_ + IP_5_ content and the consequent presence of inositol mono-and di-phosphates (IP and IP_2_) can lead to an increased mineral dialysability [35]. Recently, beneficial properties such as anticancer, antioxidative and anticalcification activities were also reported for IP_6_ and lower phosphorylated inositol phosphates, [26,36,37].

### 2.3. Polyphenols

The average concentration of phenolic acids in rapeseed meal, as obtained after analyses of the different lots collected at the rapeseed processing plant from July 2018 to June 2019, is reported in Table 3. 

The sum of the concentration of all detected phenolic compounds accounted for about 400 mg per kg of rapeseed meal (range of values detected in the different lots 395–437 mg kg^−1^). The most abundant phenolic compound of rapeseed meal was sinapic acid, which represented more than 85% of all quantified phenolic compounds (mean value 357 ± 13 mg kg^−1^, range 339–379 mg kg^−1^). Protocatechuic, 4-hydroxybenzoic and ferulic acids (mean values 14.6, 13.8 and 10.8 mg kg^−1^, respectively) accounted together for almost 10% of the total phenolic acids in rapeseed meal. The values detected in the different examined lots showed a low variability, indicating a stable quality of rapeseed and standardized conditions at the seed processing plant. 

These results are in line with values present in literature, where hydroxycinnamic acid derivatives are reported to be the most abundant bioactive compounds in rapeseed, with sinapine, a bound form of sinapic acid with anticancer, neuroprotective, antioxidant and hepatoprotective properties, as the prominent one [38]. Sinapic acid is reported to account for 70–85% of total free phenolic acids in rapeseed meal and up to 99% of phenolic acids occurring as esters and glucosides [39,40]. 

The high presence of phenolics in rapeseed meal (about 5 times higher than in its major competitor, soybean meal), limit protein digestibility and bioavailability when used in animal feeds. The presence of phenolic compounds, impacting the colour, taste and flavour, is also one of the limiting factors to the use of rapeseed protein and their derivatives in feed/food industry, if not appropriately removed [24]. Therefore, suitable technological processes need to be applied in order to reduce phenolics presence in extracts and effectively separate proteins from phenolics.

On the other side, rapeseed meal may be viewed as a sustainable source of antioxidants. Pohl et al. [41] showed antioxidant/radical scavenging properties of rapeseed meal extracts and synergistic effects of the many compounds of phenolic nature present therein. Yates et al. [38] demonstrated the value added by rapeseed meal as a source of sinapine, a bioactive molecule with antioxidant and pharmacological properties that may be extracted and used to develop nutraceutical, food or pharma products. In particular, as acetylcholine esterase inhibitor, sinapine has the focus of attention in the study of natural substances useful in the treatment of neurodegenerative diseases [42]. 

Therefore, rapeseed protein fractionation and phenolics recovery is a possible approach pursuing the double goal of feed/food performance improvement and valorization of phenols as antioxidant ingredients for various applications in high-value markets. 

### 2.4. Lipid Fraction Profile: Fatty Acids and Unsaponifiables

In spite of the oil extraction process applied upstream, a small but variable amount of fat (1.4–3.3%) was still present in rapeseed meal collected in this study. The qualitative profile of residual fat in rapeseed meal has been analyzed. 

The fatty acid profile of the lipid extract obtained from the different lots of rapeseed meal analyzed is reported in Table 4. The profile was that of rapeseed oil, with oleic acid as the major fatty acid (51–57% of total fatty acids), followed by linoleic (28–29% of total fatty acids), palmitic (9–11% of total fatty acids) and α-linolenic (4–5%) acids. Erucic acid (C 22:1 n-9), a fatty acid of toxicological concern present in rapeseed, was not detected in any of the rapeseed meal samples, as the meal originated from low-erucic acid rapeseed cultivars.

Besides a nutritionally valuable fatty acid profile, rapeseed is known to contain fat-soluble bioactive components such as sterols and tocopherols, associated with low risks of cardiovascular diseases and cancer [13]. The levels of tocopherols, tocotrienols, sterols and squalene in the different lots of rapeseed meal sampled in this study are reported in Table 5. Plant sterols were present at low levels in this side stream (average value 133 mg kg^−1^ rapeseed meal), with a variability among lots reflecting the lipid content (75–171 mg kg^−1^). Beta-sitosterol was the prevalent sterol (average 58% of total sterols), followed by stigmasterol + campesterol (22%), brassicasterol (16%), minor amounts of sitostanol (2.6%) and traces of ergosterol (≤0.5 %). Squalene was detected at very low levels (average value 1.2 mg kg^−1^). These compounds are those described in literature as prevalent in rapeseed oil, with the exception of ergosterol. The low levels of this sterol (0.47–1.04 mg kg^−1^) indicate a slight presence of molds not impairing the quality and safety of products [43,44]. In fact all necessary measures to prevent a mold contamination were taken during the production and distribution chain. Mycotoxin test analyses conducted in the frame of this project and out of the focus of this paper failed to detect any substance. 

The total tocols content of rapeseed meal was low, with an average value of about 16 mg kg^−1^ and values detected in the range 6–33 mg kg^−1^. Tocopherols were the prevalent class (98%) with values highly variable among rapeseed lots, reflecting their lipid content. The absence of β-tocopherol and β-tocotrienol in rapeseed reported in literature [13,45] allows us to reliably express data from β + γ peaks as belonging to the γ-homologue alone. Alpha-tocopherol (54% of total tocols) and γ-tocopherol (38%) were the prevalent homologues in rapeseed meal, while δ-tocopherol and tocotrienols were detected in trace amounts or not detected at all. It must be specified that the amounts reported for α-tocopherol and γ-tocopherol may be an overestimation because of the presence of unidentified coeluting compounds, as evidenced by the UV-spectrum analysis of the two peaks (not shown). 

### 2.5. Antioxidant Activity

**Figure 2:**
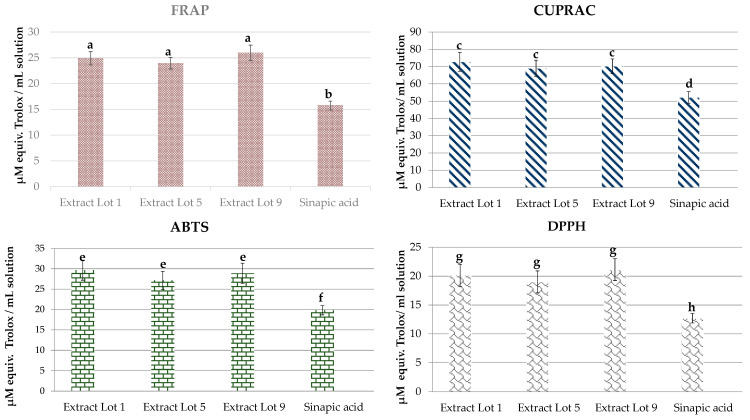
Antioxidant (FRAP, CUPRAC, ABTS, DPPH) activities of ethanolic extracts from RSM lots. Solutions normalized to 1% polyphenols, compared with the solution of 0.85% sinapic acid. The values presented represent means  ±  standard errors (*n*  =  5). Columns labeled with different letters within each type of antioxidant activity are significantly different at *p* < 0.05.

The antioxidant activity of ethanolic extracts from RSM lots compared to a 0.85% sinapic acid solution is reported in Figure 2. RSM extracts showed a higher antioxidant activity than that of sinapic acid alone, most probably due to the synergic interaction of sinapic acid with other hydroxycinnamic acids present in the RSM extract.

**Figure 3:**
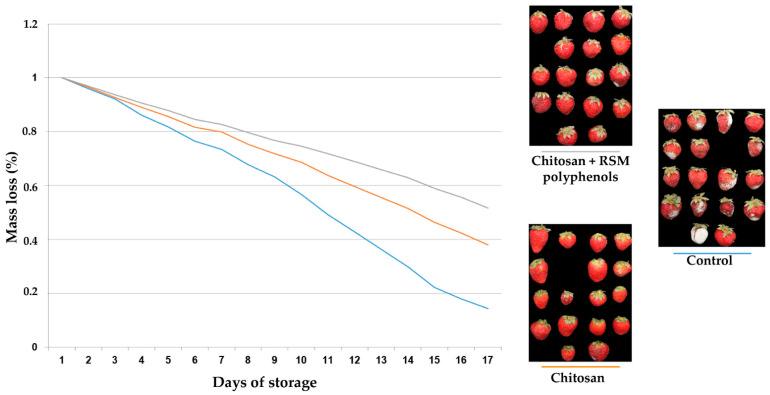
Effect of RSM polyphenols on the protective effect of chitosan on strawberry fruits.

Figure 3 presents the mass loss of strawberry fruits from different treatments. Chitosan treatment reduces the mass loss of strawberries during storage. RSM polyphenol extract enhances the protective effect of chitosan. The percentage of the decayed fruits also demonstrated the effect of RSM polyphenols on improving the protective effects of chitosan. This was reduced by 10.4% by applying the RSM polyphenols on the 17th day of the experiment. 

A similar enhancing effect on the protective effect of chitosan on strawberry fruits was recently reported for the polyphenols extracted from apple peel [46]. 

Our assay demonstrated that RSM-extracted polyphenols are primarily from phenolic acids/hydroxycinnamic acids class. These compounds have significant antioxidant activity. Such antioxidant activity could be valorized in various applications—e.g., for cosmeceuticals [47] or nutraceuticals/dietary supplements [48]. The cosmeceutical applications result from the antioxidant activity and anti-tyrosinase, anti-collagenase, anti-hyaluronidase, photoprotective, anti-inflammatory, and antimicrobial activity [47,49]. The UV-filtering and anti-radical properties of sinapate derivatives suggest an interesting potential application of sinapic acid in sunscreen formulations, replacing currently-used synthetic UV-filtering agents, shown to have a negative impact on the environment and human health [50]. This potential high-value application of sinapic acid opens new perspectives to the market segment of environmentally-friendly cosmetic products. The nutraceutical applications are not related only to the antioxidant activity. Other hydroxycinnamic bioactivities, such as anti-inflammatory [51], anti-diabetic [52], anti-hypertensive [53], neuroprotective [54], and prebiotics [55] activities are of interest for nutraceuticals / dietary supplements. Despite their proven biological activities, in general, phenolic acids / hydroxycinnamic acids are underutilized by the health and well-being industries. A limited number of patents related to hydroxycinnamic and hydroxycinnamic derivatives applications on these domains were filled so far [56,57]. The correlation with the valorization of RSM protein should boost the applications of RSM polyphenols/hydroxycinnamic acids.

In an integrated biorefinery model of RSM, an initial extraction of polyphenols, followed by their valorization as antioxidants, benefits further biorefining processes related to protein extraction by reducing the adverse effects of polyphenols on the technological and nutritional properties of rapeseed proteins [12].

## 3. Materials and Methods

### 3.1. Rapeseed Origin

Non-genetically modified rapeseed (*Brassica napus* L. var. Napus) was the core feedstock of the meal investigated in this study. Rapeseed was grown during the 2017/2018 crop season in central and east European regions, namely Slovakia, Poland, Hungary, Czech Republic, Romania and Ukraine. The seed quality met the STN 462300-1 and 2 and the Codex Alimentarius requirement of the Slovak Republic, Government Regulation no. 439/2006 and the requirements set out in the list of permitted varieties.

### 3.2. Rapeseed Meal Production and Quality Check at the Factory

Rapeseed meal was the side product of an oil-pressing factory (Poľnoservis a.s., Leopoldov, Slovakia) serving an adjacent biofuel biorefinery. The meal was a loose-solid, free-flowing material without sintered pieces over 10 cm meeting the legal requirements of the Slovak Government Regulation no. 438/2006, Act 271/2005 Coll., the European Parliament and Council Regulation (EC) no. 767/2009 and the Commission Regulation no. 68/2013 of the Fodders Catalogue. 

**Figure 4:**
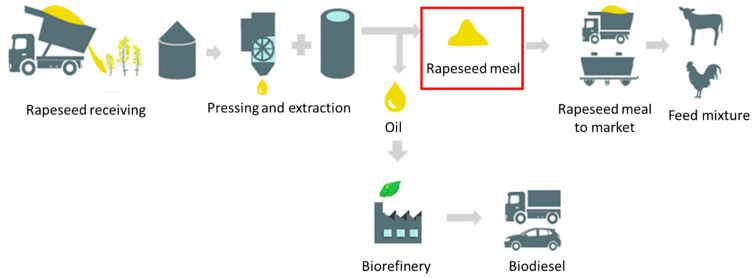
Scheme of rapeseed meal production and processing and its connection to feed market and biofuel biorefineries.

Rapeseed meal samples were collected as a by-product from the crude oil production after the oil pressing and subsequent hexane extraction process (Figure 4). The meal was allowed to cool down, put into clean plastic containers and kept in a dry place, at room temperature, avoiding heat and light sources. 

Samples were immediately taken to the laboratory for *in-house* daily routine analyses without any further treatment and analyzed within the same day of sampling. Water, dry matter, crude fat, crude protein and ash contents were determined according to the methodologies set out by the Decree of the Ministry of Agriculture of Slovak Republic 1497/4/1997-100, and checked for compliance with the internal and external limits prior to delivery for its feed purpose. This is connected to the defined conditions of rapeseed meal processing after each extraction step. All analyzed data were compared with those obtained at the CREA Research Centre for Food and Nutrition (Table 1) and served as a check that the rapeseed meal samples were not degraded/altered during storage/transportation. The quality profile of RSM was completed by accredited lab analyses according to the Government Decree of Slovak Republic No. 438/2006 relating to microbiological parameters in animal feed and content of undesirable substances and other indicators of safety and availability of feed [58]. Physical and chemical tests including parameters such as crude fiber, n-hexane content as well as heavy metals content, glucosinolates, aflatoxins, pesticides, etc. were also monitored. Results of measurements (aflatoxin B1, Cadmium, Mercury, Lead, and fluoride) complied with the requirements stated in the Decree 438/2006. Monitored results of residue levels of pesticides in feed of plant origin fully complied with Regulation (EC) No 396/2005 of the European Parliament [59]. The meal complied also with the requirements of the European Committee for Standardization (CEN) for microbiological parameters (*Enterobacteriaceae* ISO 21528-2, *Salmonella* spp. ISO 6579-1 and moulds ISO 21527-2, [60,61,62]). A monitoring study of mycotoxin and pesticide residues in rapeseed meal was also part of the same project. Results, beyond the scope of this paper, did not highlight any issues of concern. 

### 3.3. Rapeseed Meal Sampling

Once each 30–40 days, in the period July 2018–September 2019, a total of 10 samples of rapeseed meal (1 kg each) was delivered to CREA Research Centre for Food and Nutrition (Rome, Italy) for chemical analyses (Table S1). 

Upon arrival at CREA laboratories, the meal was kept refrigerated (+4 °C) until analyses. Just before analyses, rapeseed meal was milled with a lab knife-homogenizer (Grindomix GM 300, Retsch, Verder Scientific Srl, Pedrengo, Italia) until all particles passed through a 1 mm sieve and analyzed without any other processing. Proximate composition, nonprotein nitrogen, total dietary fiber, mineral content, fatty acid profile, phenolic acid profiles and unsaponifiables contents were the parameters studied. 

### 3.4. Chemicals

Pure standards of phenolic acids, tocopherols, ergosterol, stigmasterol, campesterol, β-sitosterol and squalene, were purchased from Sigma-Aldrich Inc. (St. Louis, MO, USA). Brassicasterol and sitostanol were from AVANTI Polar Lipids Inc. (Alabaster, AL, USA). Tocotrienols were purchased from Cayman Chemical Company (Ann Arbor, MI, USA).

All solvents were of analytical or high-performance liquid chromatography (HPLC) grade as required. Potassium hydroxide and trichloroacetic acid were from Carlo Erba. Tert-butyl-hydroquinone (TBHQ) was from Fluka Chemie AG (Buchs, Switzerland). Boron trifluoride in methanol (BF_3_) was from Merck (Darmstadt, Germany).

Deionized water was provided by an Arium^®^ pro UV Water Purification System (Sartorius Stedim Biotech GmbH, Goettingen, Germany). 

The reagents used for antioxidant activity determinations were purchased from Sigma-Aldrich (Merck Group, Darmstadt, Germany). The optical density was determined by a multi-mode plate reader (CLARIOstar Plus, BMG Labtech, Ortenberg, Germany).

### 3.5. Analytical Methods

#### 3.5.1. FTIR-ATR Analysis

The FTIR spectra were recorded on a Nicolet iS20 FT-IR spectrometer equipped with a diamond crystal cell for ATR operation (Thermo-Fisher Scientific, Waltam, MA, USA). The acquisition of spectra was carried out by 32 scans per sample or background in the range of 4000–650 cm^−1^ at a nominal resolution of 4 cm^−1^

The spectrum of air was used as background. The analysis was carried out at room temperature. For each measurement a lyophilized sample was placed onto the surface of the ATR crystal. Before acquiring a spectrum, the ATR crystal was cleaned with wet cellulose tissue and dried by means of a flow of nitrogen gas. The cleaned crystal was checked spectrally in order to ensure that no residue was retained from the previous sample. The spectrum of each sample was collected 10 times to check the reproducibility and make a statistical analysis. 

The FTIR spectra were analyzed with respect to the spectral band positions for identification of the signatures of the main functional groups. An assignment of the main bands was carried out by analyzing the acquired spectra and by comparing them with those present in scientific literature.

#### 3.5.2. Macronutrients and Minerals

Moisture, crude protein, crude fat and ash contents were determined following the methods of the Association of Official Analytical Chemists [63]. The crude protein content was evaluated by the Kjeldahl procedure, using 6.25 as a nitrogen-to-protein conversion factor. Nonprotein nitrogen (NPN) was determined by the Kjeldahl method after protein precipitation with 10% (*w*/*v*) trichloroacetic acid and filtration. The crude fat content was determined by Soxhlet extraction. The ash content was determined gravimetrically after incineration in a muffle furnace at 550 °C. Total dietary fiber and soluble and insoluble fiber fractions were determined according to Prosky et al. [64]. Carbohydrates were calculated by difference. All macronutrients analyses were performed in triplicate. 

Macro-minerals and trace elements (Ca, Mg, Na, K, P, Fe, Zn, Cu, Mn) were quantified by inductively coupled plasma optical emission spectrometry (Optima 8000^™^ ICP-OES, Perkin-Elmer, Waltham, MA, USA) after liquid ashing in a microwave digestion system (1200 Mega, Milestone srl, Sorisole, Italy). Mineral analyses were performed in quadruplicate.

#### 3.5.3. Phenolic Compounds 

Polyphenols were extracted from crude rapeseed meal following the procedure described by Pacifico et al. [65] with few modifications. Briefly, 200 mg of sample was weighted in a 2 mL Eppendorf tube. An amount of 1.5 mL of extraction solution (methanol/water/acetic acid, 80:19.5: 0.5, *v*/*v*/*v*) was added to the sample, vortexed for 30 s and shaken at 1000 rpm for 30 min at 4 °C (Thermomixer Cryo Comfort, Eppendorf AG, Hamburg, Germany). The samples were then centrifuged at 10,000× *g* for 10 min at 4 °C. Supernatant was collected in 5 mL volumetric flasks. This procedure was repeated 3 more times with 1 mL of extraction solution. The extract was brought to volume with extraction solution, filtered through 0.22 µm syringe filter and injected in the LC-MS/MS system. The extractions and analyses were conducted on three replicates per sample. 

Phenolic acids were quantified on an Agilent 1200 LC coupled to an Agilent 6410 triple quadrupole mass analyzer equipped with an ESI operated in negative mode. Gas temperature was 350 °C, at a flow of 11 L min^−1^. The nebulizer pressure was set at 15 psi, while the capillary voltage was 4000 V. Detection was conducted on dynamic Multiple Reaction Monitoring (dMRM) mode with the compound specific parameters displayed in Table S2. Separation was performed on a 100 mm × 1 mm Ascentis C18 column (3 μm particle size) (Supelco, Bellefonte, PA, USA) maintained at 30 °C. Mobile phase was 0.05% acetic acid in water (A) and 0.05% acetic acid in acetonitrile (B). Elution of phenolic acids was performed on gradient mode: starting at 2% B, 11.5 min 25% B, 20 min 27% B, 21 min 30% B, 25 min 30% B, 27 min 2% B, 35 min 2% B, post run time 20 min. Flow was 100 μL min^−1^ and injection volume was 1 μL. Quantification was performed according to a calibration curve constructed by injecting standard solutions of phenolic acids in the same chromatographic conditions, in a concentration range from 25 to 2000 ng mL^−1^. The lower or upper points of the curve were removed according to the concentration of each phenolic acid in the sample before quantification.

#### 3.5.4. Total Lipids and Fatty Acids 

Total lipids were extracted from rapeseed meal (about 20 g) using Ultra-Turrax T25 (Kanke & Kunkel, IKA-Labortechnik) set at 8000 rpm, with methanol, chloroform and water to keep the final proportion among the three solvents to 2:2:1.8 (*v*/*v*/*v*), in accordance with the method of Bligh and Dyer [66]. Fatty acids in the extract were methylated using boron trifluoride in methanol (BF_3_) as the esterification reagent [67]. In brief, about 25 mg of lipids were mixed with 2 mL BF_3_-methanol 12% *w*/*w* and heated in a water bath at 60 °C for 5 min. After cooling, 1 mL water and 1 mL hexane were added, then centrifuged and the upper hexane layer was transferred to a GC vial. Chromatographic separations of fatty acids were achieved on a Mega-wax column (30 m × 0.32 mm inner diameter, 0.25 μm film thickness). The esterified fatty acids were identified by means of gas-chromatography-mass spectrometry (GC-MS) and quantified by GC-Flame Ionization Detector (FID) (GC 7890A Series coupled with Mass Detector 5975C, Agilent Technologies Santa Clara, CA, USA). Fatty acids were identified by the comparison of retention times with known authentic standards and using the NIST08 Mass Spectral Library (National Institute of Standards and Technology, Gaithersburg, MD, USA). FAME Mix C4-C24 (Supelco, Bellofonte, PA, USA) was analyzed as a control of the accuracy of the analyses. Analyses were performed in triplicate. Data are reported as percent of total fatty acids.

#### 3.5.5. Unsaponifiable Lipid Fraction

Lipid extracts were also analyzed for tocopherols, tocotrienols, plant sterols and squalene contents. Analytes were quantified by High Performance Liquid Chromatography (HPLC) after saponification in ethanolic potassium hydroxide (10% *w*/*v*) using a 1100 Series Agilent HPLC System (Agilent Technologies, Santa Clara, CA, USA) equipped with a quaternary pump, solvent degasser, column thermostat and photodiode-array detector (DAD). Separation occurred on an Ultrasphere C-18 column (25 cm × 4.6 mm inner diameter, 5 m, Beckman, Palo Alto, CA, USA) coupled with a C18 guard column (15 cm × 4.6 mm, 5 µm). Chromatographic conditions were as described previously [8]. To improve the identification of compounds, rapeseed meal extracts were also analysed with an LC-MS/MS system (Agilent 1200 quaternary pump) coupled to a 6410 series triple quadrupole. The ion source was an APCI operated in positive mode. Chromatographic separation was carried out on an ACME C18-120A, 100 mm × 2.1 mm column, with 3 µm particle size. Separation conditions and ionization parameters were as previously described [8]. The concentration of sitostanol in the samples (Lot 2, 4 and 6) was measured by LC-MS/MS with the triple quadrupole analyzer operated in the MRM mode, the precursor ion 399.7 (corresponding to the molecular weight with the loss of a water molecule and an H^+^ gain) and the following product ions: 95.1 for quantification, 135.1, 109.1 and 81.1 for identification. In all cases the collision energy was 29 V, with the exception of transition 399.1 → 81.1, where it was set at 45 V. Quantification was performed by interpolation on a calibration curve constructed with pure sitostanol standard analyzed in the same conditions, in the range of 0.04 to 1.0 µg mL^−1^.

#### 3.5.6. Antioxidant Activity

The antioxidant activity of the polyphenols extracted from rapeseed meal was determined by several chemical methods (FRAP, ABTS, DDPH, CUPRAC) and by a bioassay based on enhancing edible polymer film effects on strawberry shelf life. The antioxidant activity assays were done using a solution resulted from RSM extraction in 50% ethanol. The extraction was done in ratio 1 g RSM to 20 mL ethanol solution, for 4 h at room temperature, under agitation (50 rpm). The 50% ethanolic extract solutions were normalized at 1% polyphenols, according to the Folin-Ciocâlteu method for polyphenols determination [68], using sinapic acid as a standard. The standard for antioxidant assays was a solution of 0.85% sinapic acid in 50% ethanol. The antioxidant assays were done for the ethanolic extracts from three RSM lots: Lot 1, Lot 5, and Lot 9.

The FRAP method, measuring the antioxidants’ ability to reduce a transparent tripiridiltriazine-Fe^3+^ (Fe(III)-TPTZ) complex to a blue-colored tritridiltriazine-Fe^2+^ (Fe(II)-TPTZ) complex, was accomplished according to Benzie and Strain [69]. Results were expressed as equivalent to µM Trolox per mL sample.

The antioxidant method of reducing cupric ion (CUPRAC) was carried out according to a method adapted from Meng et al. [70]. Briefly, 10 μL of sample/standard solutions of different concentrations, 30 μL CuS0_4_ (5 mM), neocuproin 30 μL (3.75 mM) and 280 μL of distilled water were mixed, reaching a final volume of 350 μL. After 30 min, the optical density was read at λ = 450 nm in a multi-mode plate reader. The standard solutions started from a stock solution of 10 mM Trolox and were used for calibration curve concentrations: 0–2 mM. The results were expressed as equivalent to µM Trolox per mL sample.

Scavenging the ABTS radical cation was also used to determine the antioxidant activity of RSM polyphenols extracts [71]. Trolox was used as a standard antioxidant substance, the calibration curve being made in the concentration range 0–40 μM, starting from a stock solution of Trolox 1 mM. The results were expressed as µM Trolox equivalent per mL sample. The ABTS^•+^ scavenging capacity of the RSM polyphenolic extract and Trolox were calculated according to the following Formula (1):

(1) ABTS Scavenging activity=ODcontrol−ODsampleODcontrol

where *OD_control_* is the optical density of ABTS radical in ethanol; *OD_sample_* is the absorbance of ABTS radical solution mixed with RSM polyphenol extract, sinapic acid ethanol solution, or Trolox standard.

The measurement of antioxidant activity of RSM polyphenols extracts was also done by scavenging the DPPH^•^ radical [72]. The reaction mixture consisted of a 100 μL sample/standard and 100 μL of 0,3 mM DPPH radical solution in ethanol. The reading of the absorbents was carried out at λ = 517 nm after 30 min of reaction using a UV-VIS multi-mode plate reader. Trolox was used as a standard substance, the calibration curve being made in the concentration range of 0.15 mM–0.0125 mM, starting from a stock solution of Trolox 1 mM. The results were expressed as µM Trolox per mL sample. The DPPH^•^ scavenging capacity of the RSM polyphenolic extract and Trolox were calculated according to the following Formula (2):

(2) DPPH• Scavenging activity=ODcontrol−ODsampleODcontrol

where *OD_control_* is the optical density of DPPH^•^ radical in ethanol; *OD_sample_* is the absorbance of ABTS radical solution mixed with RSM polyphenol extract, sinapic acid ethanolic solution, or Trolox standard.

The strawberry shelf-life bioassay was developed based on the proven effects of polyphenols antioxidants on the efficiency of polymer coatings in prolonging strawberries’ shelf life [73]. The surface of the strawberry fruits (*Fragaria × ananassa*, cv. Alba) was disinfected by immersion in a sodium hypochlorite 1% solution for 2 min, followed by repeated rinsing with sterile water and slow drying. Chitosan solution was prepared by dissolving fungal chitosan into the acetic acid solution. The fungal chitosan was isolated from *Ganoderma lucidum* [74], and the average molecular weight and degree of deacetylation were 209 kDa and 86%, respectively. The strawberries were coated with 1% chitosan solution and 1% chitosan with 1% polyphenols RSM extract. The coating was done by dipping for 30 s in the chitosan solution and chitosan with RSM polyphenols, respectively. The coating conditions applied were selected after preliminary studies indicating these as the best ones. The treated strawberry fruits were drained in a tray with holes and dried for 2 h at ambient temperature. The treated strawberries and the untreated strawberries (control) were stored in polypropylene plastic trays, at 20°C and 35–40% RH for 17 d to test the effect of the combined treatment on the storage at room conditions in stressful conditions for treated strawberry fruit.

The following parameters were monitored during the storage period: mass loss (%) and percentage of the decayed strawberry fruits. Mass loss was calculated according to the following Equation (3):

(3) Mass loss=Mi−MoMi

where *M_i_* is the initial mass of the strawberry and *M_o_* is the mass determined in each day from the experiment. The percentage of decayed fruits was defined as the percentage ratio of decayed fruits (visually inspected) per total number of fruits included in the experiment.

#### 3.5.7. Quality Assurance 

For sterols, tocols and squalene determination, the concentrations of stock solutions of pure standards were determined spectrophotometrically using their specific absorption coefficients. For each analyte, external linear calibration curves of analytical standards, with a minimum of five concentration levels, were constructed. Within the calibration ranges, the DAD response for each analyte was linear and the correlation coefficient exceeded 0.998. Repeatability was estimated by calculating the coefficient of variation (CV) after repeated runs of a standard solution containing each compound at the level found in samples. The purity of the peaks was checked by matching the UV/Vis spectra of each peak with that of the related standard molecule. For validation of the method and quality control of tocopherol data, the standard reference material NIST 3278 (Tocopherols in edible oils) was analyzed. For validation of the method and quality control of minerals and trace elements data, two Certified Reference Materials, cabbage (IAEA-359, International Atomic Energy Agency Reference Materials Group) and haricots vert (BCR 383, Community Bureau of Reference, Brussels) were analyzed. All analyses were performed at least in triplicate.

#### 3.5.8. Data Treatment 

For each parameter, analyses of individual lots of rapeseed meal were performed at least in triplicate. Data for single lots were reported as mean, standard deviation and range of values detected during the experimental period. Analysis of variance (One-Way ANOVA) and Multiple-Range LSD test were applied to find out significant differences (*p* ≤ 0.05), if any, among the antioxidant activity of RSM phenol extracts and sinapic acid standard solution (Statgraphics^®^, Statistical Graphic System by Statistical Graphic Corporation, Version 5 Plus, Manugistics^™^ Inc., Rockville, MD, USA).

## 4. Conclusions

This study highlights the potentialities of rapeseed meal as a candidate feedstock for further valorization via subsequent biorefining processes. The characterization of bioactive components profile together with the qualitative analysis by mid infrared spectroscopy can represent a useful database to validate and make the FTIR technique a reliable tool for quality monitoring of active principles in rapeseed meal. 

Although safety aspects are out of the focus of this paper, they were considered in the frame of this study, since a constant monitoring of safety parameters throughout the production and distribution chain, as well as at a biorefinery level, is of primary importance to exclude the presence of contaminants in a biomass intended to be used for final feed-food-cosmetic applications. 

The results of this study indicate proteins and phenolics as the components with high valorization potential for further biorefining of this oilseed extraction co-product connected to the agri-food and biofuel sectors. Integrated biorefinery systems could open new perspectives to the utilization of rapeseed meal, traditionally used as animal feed. The variability is relatively low for proteins and polyphenols in analyzed rapeseed meal samples. Therefore, a biorefinery industrial process for recovery of proteins and polyphenols could be easily standardized. At the same time, the creation of new bio-based value-chains and establishment of new cross-sector interconnections could improve the economics and sustainability of all bioeconomy sectors involved in oilseed management.

Apart from the advantages connected to their sustainable origin, rapeseed proteins and derivatives have potentialities in the food and nutraceutical sectors as substitutes of conventional proteins in products targeted to vegans or consumers allergic to milk/egg proteins or intolerant to gluten. On the other side, polyphenols, and particularly sinapic acid, have a high potential for valorization in nutraceuticals and cosmetic products, for example as ingredient in environmentally friendly sunscreen products. Overall, proteins and phenolics from rapeseed meal have the potential of platform molecules suitable for various applications in high-value markets (food, cosmetics, nutraceutics), urged by innovation and sustainability needs in a context of declining resources.

An efficient separation of proteins from the other components, in order to have ingredients with the highest functional and sensory properties, is one of the main technical challenges for a full valorization of this side stream. In the frame of a zero-waste circular economy approach, the implementation of feasible and effective cascade processes to recover proteins and phenolics separately and the assessment of related techno-economical and sustainability evaluations represent the next steps of this project. 

## Figures and Tables

**Table 1:** Chemical composition of rapeseed meal samples. Mean, standard deviation and range of values obtained from different lots collected between July 2018 and September 2019 (*n* = 10) *.

	Lot 1	Lot 2	Lot 3	Lot 4	Lot 5	Lot 6	Lot 7	Lot 8	Lot 9	Lot 10	Mean	sd	Min	Max
pH	-	6.17	5.84	6.16	6.39	6.07	6.24	6.10	6.02	6.06	6.12	0.15	5.84	6.39
	g kg^−1^													
Dry matter	871.60	874.47	876.97	893.50	866.53	880.23	873.43	866.87	874.50	872.90	875.10	7.68	866.53	893.50
Water content	128.40	125.53	123.03	106.50	133.47	119.77	126.57	133.13	125.50	127.10	124.90	7.68	106.50	133.47
Total N	50.93	52.57	54.27	54.07	51.27	51.83	53.77	48.90	48.50	49.88	51.60	2.09	48.50	54.27
Crude protein	318.40	328.55	339.13	337.95	320.27	324.09	336.02	305.43	302.98	311.76	322.46	13.11	302.98	339.13
Nonprotein N	3.18	3.77	5.54	5.54	3.68	4.94	4.18	3.83	5.01	4.21	4.39	0.82	3.18	5.54
Ash	68.00	56.83	54.83	58.07	58.33	57.60	55.00	56.43	62.63	64.30	59.20	4.34	54.83	68.00
Crude Fat	31.90	25.27	14.23	17.33	33.33	18.36	14.13	17.17	19.73	21.97	21.34	6.82	14.13	33.33
Total dietary fiber	371.93	400.77	368.30	368.50	337.30	340.00	334.90	362.35	342.50 ^a^	315.00 ^b^	354.16	24.69	315.00	400.77
Carbohydrates	81.37	62.64	97.53	111.01	118.22	139.42	133.64	122.84	145.25	158.52	117.04	29.77	62.64	158.52

* data for each lot represent mean of triplicate measurements. Details on the origin of rapeseed meal and on lot sampling are provided in Section 3 and in Table S1. ^a^ Lot 9: Soluble fiber 43.5 ± 6.4 g kg^−1^, Insoluble fiber: 299.0 ± 9.9 g kg^−1^. Total dietary fiber = 12.7% soluble, 87.3% insoluble. ^b^ Lot 10: Soluble fiber 41.0 ± 9.9 g kg^−1^, Insoluble fiber: 274.0 ± 7.1 g kg^−1^. Total dietary fiber = 13.0% soluble, 87.0% insoluble.

**Table 2:** Mineral and trace element contents of rapeseed meal samples. Mean, standard deviation, and range of values obtained from different lots collected between July 2018 and September 2019 (*n* = 10) *.

	Lot 1	Lot 2	Lot 3	Lot 4	Lot 5	Lot 6	Lot 7	Lot 8	Lot 9	Lot 10	Mean	sd	Min	Max
	g kg^−1^													
K	10.57	10.44	11.12	10.87	10.14	11.45	9.84	10.01	11.45	13.28	10.92	1.01	9.84	13.28
P	8.98	9.05	8.29	8.82	8.89	8.36	8.22	8.19	8.68	9.43	8.69	0.42	8.19	9.43
Ca	6.08	6.24	5.75	6.80	6.51	6.62	6.24	6.74	7.33	6.82	6.51	0.45	5.75	7.33
Mg	3.64	4.12	4.03	4.69	4.38	4.49	4.28	4.17	3.50	3.86	4.11	0.37	3.50	4.69
Na	1.15	1.15	0.15	0.03	1.03	0.14	0.05	0.04	0.09	0.06	0.39	0.50	0.03	1.15
	mg kg^−1^													
Fe	88.40	101.35	78.10	106.61	89.14	86.59	95.35	78.19	69.23	72.91	86.59	12.19	69.23	106.61
Zn	52.76	52.71	55.48	53.68	49.40	49.20	55.57	49.85	49.36	57.13	52.52	2.95	49.20	57.13
Mn	52.69	62.26	52.99	60.99	56.96	56.93	54.70	54.68	37.77	45.68	53.56	7.22	37.77	62.26
Cu	3.77	5.33	4.31	3.25	3.62	3.42	4.00	3.89	3.26	4.83	3.97	0.68	3.25	5.33

* Values are reported on a dry mass basis, as received from the processing plant (11–13% moisture). Data for each lot represent the mean of quadruplicate measurements. Details on the origin of rapeseed meal and on lot sampling are provided in Section 3 and in Table S1.

**Table 3:** Concentration of phenolic acids in rapeseed meal samples and sum of all detected phenolic compounds (mg kg^−1^ product). Mean, standard deviation, and range of values obtained from different lots collected between July 2018 and July 2019 (*n* = 8) *.

	Lot 1	Lot 2	Lot 3	Lot 5	Lot 6	Lot 7	Lot 8	Lot 9	Mean	sd	Min	Max
	mg kg^−1^											
Gallic acid	1.41	1.44	1.60	1.43	1.62	1.59	1.78	1.63	1.56	0.13	1.41	1.78
Protocatechuic acid	13.25	15.10	14.66	14.12	16.02	13.42	15.23	15.14	14.62	0.96	13.25	16.02
4-Hydroxybenzoic acid	13.59	12.77	14.62	14.60	13.99	13.56	14.19	12.83	13.77	0.72	12.77	14.62
Vanillic acid	3.44	2.79	3.97	3.31	3.30	3.28	3.69	3.16	3.37	0.35	2.79	3.97
Chlorogenic acid	1.96	2.17	2.08	1.90	2.07	2.08	1.98	2.02	2.03	0.09	1.90	2.17
Neochlorogenic acid	3.03	3.08	2.98	3.02	3.00	2.99	2.83	2.90	2.98	0.08	2.83	3.08
Caffeic acid	2.55	2.66	2.69	2.40	3.00	2.68	2.57	2.89	2.68	0.19	2.40	3.00
Syringic acid	2.31	1.64	2.76	2.41	2.68	2.37	3.12	2.41	2.46	0.43	1.64	3.12
Cryptochlorogenic acid	2.08	2.21	2.32	2.12	0.79	0.69	0.57	0.67	1.43	0.81	0.57	2.32
p-Coumaric acid	2.44	2.27	2.51	2.07	2.46	2.18	1.57	1.75	2.16	0.34	1.57	2.51
Ferulic acid	10.82	10.36	11.36	10.72	11.43	10.34	10.03	11.27	10.79	0.53	10.03	11.43
Sinapic acid	378.68	348.03	348.89	356.81	348.68	339.20	366.73	370.11	357.14	13.46	339.20	378.68
Cinnamic acid	0.95	1.09	0.94	1.2	1.08	1.01	0.83	1.03	1.02	0.11	0.83	1.20
Σ phenolic acids	436.51	405.62	411.38	416.12	410.11	395.38	425.11	427.81	416.01	13.27	395.38	436.51

* Values are reported on a dry mass basis, as received from the processing plant (11–13% moisture). Data for each lot represent the mean of triplicate measurements. Details on the origin of rapeseed meal and on lot sampling are provided in Section 3 and in Table S1.

**Table 4:** Fatty acid profile of rapeseed meal samples. Mean, standard deviation, and range of values obtained from different lots collected between July 2018 and July 2019 (*n* = 8) *.

	Lot 1	Lot 2	Lot 3	Lot 5	Lot 6	Lot 7	Lot 8	Lot 9	Mean	sd	Min	Max
	% of total fatty acids											
Lauric acid (C12:0)	0.00	0.02	0.01	0.06	0.06	0.05	0.00	0.00	0.03	0.03	0.00	0.06
Myristic acid (C14:0)	0.15	0.16	0.12	0.17	0.17	0.18	0.24	0.24	0.18	0.04	0.12	0.24
Pentadecylic acid (C15:0)	0.00	0.07	0.12	0.00	0.00	0.00	0.00	0.00	0.02	0.04	0.00	0.12
Palmitic acid (C16:0)	11.10	11.18	9.15	8.94	10.73	8.74	8.81	8.60	9.66	1.13	8.60	11.18
Palmitoleic acid (C16:1 n-7)	0.47	0.75	0.63	0.99	0.87	0.80	0.88	1.12	0.81	0.20	0.47	1.12
Hexadecadienoic acid (C16:2, n-4)	0.00	0.20	0.23	0.19	0.18	0.23	0.00	0.17	0.17	0.08	0.00	0.23
Margaric acid (C17:0)	0.09	0.07	0.08	0.07	0.05	0.05	0.09	0.00	0.06	0.03	0.00	0.09
Stearic acid (C18:0)	1.92	1.96	1.30	0.82	0.84	0.67	0.76	0.63	1.11	0.55	0.63	1.96
Oleic acid (C18:1n-9)	51.79	50.81	54.64	54.22	52.96	55.16	54.35	56.99	53.86	1.96	50.81	56.99
Linoleic acid (C18:2 n-6)	29.75	29.90	29.25	29.54	28.57	29.32	29.70	27.30	29.17	0.86	27.30	29.90
α-Linolenic acid (C18:3 n-3)	4.20	4.32	4.16	4.48	4.54	4.56	4.93	5.04	4.53	0.32	4.16	5.04
Arachidic acid (C20:0)	0.21	0.26	0.14	0.21	0.41	0.24	0.25	0.00	0.25	0.08	0.14	0.41
Gondoic acid (C20:1 n-9)	0.20	0.20	0.08	0.23	0.62	0.00	0.00	0.00	0.27	0.21	0.08	0.62
Eicosadienoic acid (C20:2 n-6)	0.00	0.04	0.01	0.01	0.00	0.00	0.00	0.00	0.01	0.02	0.00	0.04
Behenic acid (C22:0)	0.12	0.10	0.08	0.06	0.00	0.00	0.00	0.00	0.09	0.03	0.06	0.12
Erucic acid (C22:1 n-9)	-	-	-	-	-	-	-	-	-	-	-	-
Total SFA	13.59	13.82	11.00	10.33	12.27	9.94	10.15	9.48	11.32	1.69	9.48	13.82
Total MUFA	52.46	51.76	55.36	55.44	54.44	55.96	55.22	58.10	54.84	2.00	51.76	58.10
Total PUFA	33.95	34.39	33.65	34.23	33.29	34.11	34.63	32.42	33.84	0.71	32.42	34.63
Total n-3 PUFA	4.20	4.32	4.16	4.48	4.54	4.56	4.93	5.04	4.53	0.32	4.16	5.04
Total n-6 PUFA	29.75	29.94	29.26	29.56	28.57	29.32	29.70	27.30	29.17	0.87	27.30	29.94
n-6/n-3 PUFA ratio	7.08	6.94	7.04	6.60	6.32	6.43	6.05	5.46	6.49	0.55	5.46	7.08
PUFA/SFA ratio	2.50	2.49	3.15	3.32	2.72	3.43	3.42	3.43	3.06	0.42	2.49	3.43

* data for each lot represent the mean of triplicate measurements. Details on the origin of rapeseed meal and on lot sampling are provided in Section 3 and in Table S1. - not detected.

**Table 5:** Levels of tocopherols, tocotrienols, sterols and squalene in rapeseed meal samples. Mean, standard deviation, and range of values obtained from different lots collected between July 2018 and May 2019 (*n* = 6) *.

	Lot 1	Lot 2	Lot 3	Lot 4	Lot 6	Lot 7	Mean	sd	Min	Max
	mg kg^−1^									
α-tocopherol ^a^	18.44	8.65	7.33	3.66	6.47	7.38	8.65	5.08	3.66	18.44
γ-tocopherol ^a^	10.40	6.61	6.50	2.96	5.73	4.65	6.14	2.49	2.96	10.40
δ-tocopherol	3.10	0.00	0.00	0.00	0.00	2.06	0.86	1.37	0.00	3.10
α-tocotrienol	1.12	0.00	0.52	0.00	0.00	0.00	0.27	0.47	0.00	1.12
γ-tocotrienol	0.30	0.00	0.00	0.00	0.00	0.00	0.05	0.12	0.00	0.30
δ-tocotrienol	0.00	0.00	0.00	0.00	0.00	0.00	0.00	0.00	0.00	0.00
Ergosterol	1.01	1.04	0.70	0.47	0.53	0.47	0.71	0.26	0.47	1.04
Brassicasterol ^a^	8.14	37.16	35.06	16.98	31.75	34.19	27.21	11.82	8.14	37.16
Stigma + campesterol	74.26	35.68	30.80	14.64	32.01	33.09	36.75	19.84	14.64	74.26
β-sitosterol	172.71	92.76	91.23	39.55	83.64	102.24	97.02	43.11	39.55	172.71
Sitostanol	-	4.37	-	3.72	4.96	-	4.35	0.62	3.72	4.96
Squalene	0.51	0.47	1.84	0.45	1.19	1.53	1.17	0.56	0.47	1.84
Sum of sterols	---	171.00	---	75.36	152.90	---	133.09	50.81	75.36	171.00
Sum of tocopherols	31.95	15.26	13.83	6.62	12.20	14.08	15.66	8.54	6.62	31.95
Sum of tocotrienol	1.42	0.00	0.52	0.00	0.00	0.00	0.32	0.58	0.00	1.42
Sum of tocols	33.37	15.26	14.35	6.62	12.20	14.08	15.98	9.07	6.62	33.37

* data for each lot represent the mean of triplicate measurements. Details on the origin of rapeseed meal and on lot sampling are provided in Section 3 and in Table S1. - data not available, --- not applicable. ^a^ impure, coelution of an unidentified substance, as evidenced by the UV spectrum.

## Data Availability

The data presented in this study are available in the article and in supplementary material.

## Supplementary Materials

The following are available online. Table S1: Sampling date at Poľnoservis’s plants of the different rapeseed meal lots analyzed in this study, Table S2: Ionization and detection parameters of phenolic compounds.
