# Supplementary material for: Valorization Potentials of Rapeseed Meal in a Biorefinery Perspective: Focus on Nutritional and Bioactive Components
Source: Molecules. 2021 Nov 10;26(22):6787. doi: 10.3390/molecules26226787 (PMC8618708; doi:10.3390/molecules26226787)
Supplement: Supplementary file 1 [file molecules-26-06787-s001.zip › molecules-1442015-supplementary.pdf]

Supplementary information

Valorisation potentials of rapeseed meal in a biorefinery perspective: focus on nutritional and bioactive components

Gabriella Di Lena [1,*], Jose Sanchez del Pulgar [1], Massimo Lucarini [1], Alessandra Durazzo [1], Petra Ondrejíčková [2], Florin Oancea [3], Rodica-Mihaela Frincu [3], Altero Aguzzi [1], Stefano Ferrari Nicoli [1], Irene Casini [1], Paolo Gabrielli [1], Roberto Caproni [1], Igor Červeň [4], and Ginevra Lombardi-Boccia [1]

[1] CREA Research Centre for Food and Nutrition, Via Ardeatina 546, 00178 Rome, Italy; jsapuri@hotmail.com (J.S.d.P.); massimo.lucarini@crea.gov.it (M.L.); alessandra.durazzo@crea.gov.it (A.D.); altero.aguzzi@crea.gov.it (A.A.), stefano.nicoli@crea.gov.it (S.F.N.), irene.casini@crea.gov.it (I.C.), paolo.gabrielli@crea.gov.it (P.G.), roberto.caproni@crea.gov.it (R.C.), g.lombardiboccia@crea.gov.it (G.L.B.)

[2] ENVIRAL a.s., Trnavská cesta, 920 41 Leopoldov, Slovak Republic; Ondrejickova@enviengroup.eu (P.O.)

[3] National Institute for Research and Development in Chemistry and Petrochemistry – ICECHIM, Bucharest; florin.oancea@icechim.ro (F.O.), icechim.calarasi@gmail.com (M.F.)

[4] Poľnoservis a.s., Trnavská cesta, 920 41 Leopoldov, Slovak Republic; cerven@polnoservis.sk (I.Č.)

* Correspondence: gabriella.dilena@crea.gov.it; Tel.: +39-06-51494501

Table S1. Sampling date at Poľnoservis's plants of the different rapeseed meal lots analysed in this study.

RAPESEED MEAL	
**LOT**	**Sampling date**
**1**	2/7/2018
**2**	22/10/2018
**3**	23/11/2018
**4**	21/1/2019
**5**	25/2/2019
**6**	23/4/2019
**7**	30/5/2019
**8**	18/6/2019
**9**	29/7/2019
**10**	26/9/2019

Table S2. Ionization and detection parameters of phenolic compounds.

Compound Name	Prec. Ion	Prod. Ion	Frag.	CE	Cell Volt	Polarity
Gallic acid	169.1	125	80	9	7	Negative
	169.1	79	80	21	7	Negative
Protocatechuic acid	153.1	109	80	13	7	Negative
	153.1	108	80	25	7	Negative
4-Hydroxybenzoic acid	137.1	93	80	13	7	Negative
	137.1	65	80	33	7	Negative
Neochlorogenic acid	353.3	191	110	13	7	Negative
	353.3	179	110	13	7	Negative
Vanillic Acid	167.1	151.9	80	5	7	Negative
	167.1	108	80	17	7	Negative
Chlorogenic acid	353.3	191	80	9	7	Negative
	353.3	85	80	45	7	Negative
Caffeic acid	179.2	135	80	13	7	Negative
	179.2	134.2	80	25	7	Negative
Syringic acid	197.2	182	80	9	7	Negative
	197.2	123	80	21	7	Negative
Cryptochlorogenic acid	353.3	179	110	13	7	Negative
	353.3	173	110	13	7	Negative
p-Coumaric acid	163.2	119	80	5	7	Negative
	163.2	93	80	33	7	Negative
Ferulic acid	193.2	178	80	9	7	Negative
	193.2	134	80	13	7	Negative
Sinapic acid	223.2	208	80	5	7	Negative
	223.2	93	80	33	7	Negative
Cinnamic acid	147.2	103	80	5	7	Negative
	147.2	77	80	21	7	Negative

Prec. Ion: Precursor ion m/z; *Prod. Ion*: Product ion m/z; *Frag.*: Fragmentor potential (V); *CE*: Collision energy (V); *Cell Volt*: Cell acceleration voltage.
